# Money Value of Medical Attendance

**Published:** 1892-07

**Authors:** 


### THE MONEY VALUE OF MEDICAL ATTENDANCE.

A case of much interest has recently attracted some comment in the papers of New York and Atlanta. A wealthy gentleman of the latter city summoned Dr. Barrows from New York to attend a member of his family in a case of fever. Dr. Barrows is the assistant of Dr. W. M. Polk, and receives a salary of $3,200 a year. He was in attendance upon the case ten days, and rendered a bill for services at the rate of $250 per day. Payment was refused on the ground of exorbitant charge, and Dr. Polk brought suit. At the trial Dr. Jno. A. Wyeth testified that he would have expected to receive $300 per day in circumstances such as these. Dr. Loomis stated that he would value his exclusive services at not less than $240 per day in New York, or $500 per day if called to Atlanta. Other New York physicians testified that the charges were entirely reasonable. Drs. Kendrick and Armstrong, of Atlanta, who were also in attendance upon the case, gave affidavit that they regarded Dr. Barrows only as a consultant from New York, that he only approved their management of the case and made a few "suggestions about some minor matters," and that they regarded $50 per day as a just and ample compensation.

The jury, it is said, after much deliberation and debate, returned a verdict in the nature of a compromise, allowing Dr. Polk $1,500, or $150 per day, for the services of Dr. Barrows.

In this case all of this trouble and annoyance might have been obviated by a short telegraphic correspondence as to the matter of expense. There is an ancient proverb to the effect that he never buys anything cheap who does not first inquire the price. In the absence of such previous arrangement the gentleman who called

Dr. Barrows from his work in New York had no right to object if the latter estimated the value of his exclusive services in Atlanta in accordance with the professional standard in New York. It was not a question of the value of the time which Dr. Barrows spent *in Atlanta*, but it was the value of the time which he spent *out of New York*.

The case was an interesting and important one from the fact that it furnishes a jury's estimate of the money-value of exclusive medical services rendered in such conditions as the above.

